# Dose–response to endurance training: a longitudinal study of training load and biomarkers in elite triathletes

**DOI:** 10.3389/fphys.2026.1767378

**Published:** 2026-05-20

**Authors:** Roberto Cejuela, Héctor Arévalo-Chico, Sergio Sellés-Pérez

**Affiliations:** Physical Education and Sports, Faculty of Education, University of Alicante, San Vicente del Raspeig, Spain

**Keywords:** biomarkers, high performance, performance analysis, training load, training periodization, triathlon

## Abstract

This study examined the relationships between fatigue-related blood biomarkers, training load distribution, and performance changes in elite triathletes across a structured training season. Twenty athletes (11 international-level and 9 national-level) were monitored. Exercise stimuli were quantified using objective and subjective metrics during the general and specific preparatory periods, with each microcycle classified as heavy, moderate, or low according to accumulated weekly load. Baseline and follow-up blood samples were collected to assess urea and creatine kinase (CK), and an incremental cycling test with gas analysis was performed at the beginning and end of the training period to evaluate changes in physiological performance. Statistical analyses assessed pre–post differences, while correlation analyses explored associations between training load, biomarker responses, and performance outcomes. Urea concentrations increased significantly during severe (p < 0.001; ES: 2.1) and moderate (p < 0.001; ES: 1.2) load microcycles compared with baseline, whereas CK showed no meaningful associations across the season. Greater accumulated subjective training load was associated with larger increases in urea (ϱ = 0.67; p < 0.05), whereas a more favorable balance between the objectively monitored training load and the athlete’s perceived exertion—characterized by lower perceived effort relative to the external load—was associated with smaller urea elevations (ϱ = -0.55; p<0.05). In addition, improvements in relative power at V̇O_2_max and VT2 were negatively associated with increases in urea ((ϱ = -0.65; p < 0.05) (ϱ = -0.68; p < 0.05) respectively). Despite phases of comparable exercise stimuli, no differences in biomarker responses were observed between national- and international-level triathletes. These findings suggest that longitudinal monitoring of urea may provide useful information to help identify potential states of excessive training load in athletes. However, the substantial inter-individual variability observed indicates that isolated biomarker measurements are insufficient to identify non-adaptive responses. Therefore, an individualized, longitudinal, and holistic monitoring framework integrating objective and subjective training load measures is required to better understand training-induced adaptations and fatigue.

## 1 Introduction

Analyzing the relationship between training load and the resulting physiological responses has been a major line of research in endurance sports (Greenham et al., 2018). In this context, sports training represents a structured and systematic process in which physical loads are applied with the objective of eliciting adaptations that enhance competitive performance (Issurin, 2016). Throughout a training season, the repeated application of these exercise stimuli induces acute disturbances in physiological homeostasis, which, when appropriately managed, result in beneficial long-term adaptations (Impellizzeri et al., 2023). Monitoring training stimuli and the corresponding physiological responses in elite athletes may therefore provide valuable insights into the adaptive processes occurring across different phases of preparation and help to better characterize the psychophysiological stress imposed by training.

Several variables can be used to characterize the magnitude of the training stimulus and support coaches in the planning of training programs. These variables are commonly classified into external load, referring to the objective and measurable components of training, and internal load, reflecting the athlete’s psychophysiological responses to a given external stimulus (Impellizzeri et al., 2019). By monitoring internal and/or external load variables, several training load quantification models have been developed to assess the psychophysiological impact of the activity on the athlete. Training load monitoring, if done in a rigorous and easy-to-interpret manner, provides valuable information on the responses associated with training (Halson, 2014).

However, it is necessary for coaches to critically analyze the validity of training load measurement models according to their sport (Passfield et al., 2022). To this end, it is advisable to examine the relationships between training load metrics, specific fatigue markers, and subsequent performance improvements (Impellizzeri et al., 2023). Moreover, incorporating metrics that relate external objective training load variables to indicators of internal load (such as rate of perceived exertion) may provide additional insight into the athletes’ level of adaptation to the training stimulus (Lima-Alves et al., 2021). In this regard, the interaction between external and internal load is particularly relevant, as a given external stimulus may elicit different internal responses depending on the athlete’s physiological state, fitness level, and accumulated fatigue. Consequently, this interaction could modulate both the magnitude and direction of fatigue markers, as well as subsequent performance outcomes.

To analyze the degree of physiological stress and fatigue, blood biomarkers are usually measured (e.g., urea, cortisol or creatine kinase (CK) (Banfi et al., 2012)). Although not all of them may be sensitive to different training doses, they can provide an approximate indication of the fatigue induced by physical exercise (Halson, 2014). In endurance sports, the relationship between training load and blood urea concentrations is well documented, with increases in training volume and intensity typically associated with elevated urea levels, reflecting greater protein turnover and metabolic stress (Greenham et al., 2018; López-Chicharro and Fernández-Vaquero, 2022). Similarly, CK is widely used as an indirect marker of muscle damage, as it is released into the bloodstream following muscle fiber disruption (López-Chicharro and Fernández-Vaquero, 2022). Importantly, these biomarkers also present practical advantages, as they can be easily measured, enabling frequent and minimally invasive monitoring without interfering with athletes’ training routines.

Several studies have demonstrated associations between the application of training loads and increases in biomarker levels over short training periods or training camps (Coutts et al., 2007; Hecksteden et al., 2016; Sperlich et al., 2016; MaChado et al., 2018; Wahl et al., 2021). However, establishing relationships over long periods of training is more complex. As training periods are extended, chronic adaptations within the organism progressively modulate acute physiological responses, thereby requiring the application of higher training loads to elicit further stimuli (Cardona et al., 2019).

Furthermore, responses of certain biomarkers may be influenced by external factors such as nutrition, recovery, or mood, resulting in high inter-individual variability (Noone et al., 2024; Biljak et al., 2025). Therefore, more sensitive training load quantification models and more specific protocols are needed to accurately detect physiological variations associated with changes in training—particularly in well-trained athletes, where higher training loads are required to elicit responses (McKay et al., 2022), or during advanced stages of the training period. Haller et al. (2023), highlights the importance of specific methodological considerations for the accurate interpretation of blood biomarkers values in athletes. Key recommendations include establishing individualized baseline levels during rest periods, standardizing protocols for sample collection and timing, and integrating the interpretation of biomarkers values with contextual data such as objective external training load, subjective perception of effort, and athlete performance.

Analyzing biomarker responses in real-world training contexts can substantially enhance our understanding of the physiological adaptations to training loads and their association with subsequent performance improvements. At the same time, it offers an opportunity to validate existing training load models by linking them to objective biochemical markers, thereby enhancing both practical monitoring strategies and scientific frameworks (Passfield et al., 2022).

In the context of triathlon, this complexity increases even further due to the combination of three distinct disciplines, each of which imposes unique physiological demands on the body and can interact between them (Millet et al., 2002). As a result, training load quantification models must account for the specific stress profiles of swimming, cycling, and running. Moreover, the high training volumes and intensities typically required in triathlon can lead to extreme fatigue states, increasing the risk of over-training and overuse injuries (Vleck et al., 2014).

Also, obtaining these data in high-performance athletes can be particularly challenging, as competitive demands and dense training schedules often interfere with the timing, frequency, and consistency of measurements. Access to elite athletes is inherently limited, and data collection must be carefully integrated within their performance routines to avoid disrupting training or competition. Additionally, logistical constraints, travel, and the need to prioritize performance outcomes over research procedures can further reduce control over experimental conditions (Sandbakk et al., 2023).

The aim of this study was to analyze the relationship between different training load doses and fatigue biomarkers throughout the general and specific preparatory periods in elite triathletes. Additionally, the study sought to examine whether improvements in performance may be influenced by differences in training loads and biomarker responses, as well as to compare the dose–response process between athletes of different competitive performance levels.

It was hypothesized that increases in urea and CK levels would be associated with periods of high training load. It is also expected that athletes exhibiting lower perceived fatigue relative to the administered training load will show greater performance improvements following the training period, along with smaller increases in urea and CK levels. Finally, it was hypothesized that higher-level triathletes would accumulate higher training loads compared to national-level athletes; however, no significant differences in biomarker responses or performance improvements between the two groups were expected, assuming that each athlete followed training plans tailored to their individual characteristics.

## 2 Materials and methods

### 2.1 Study design

**Figure 1:**
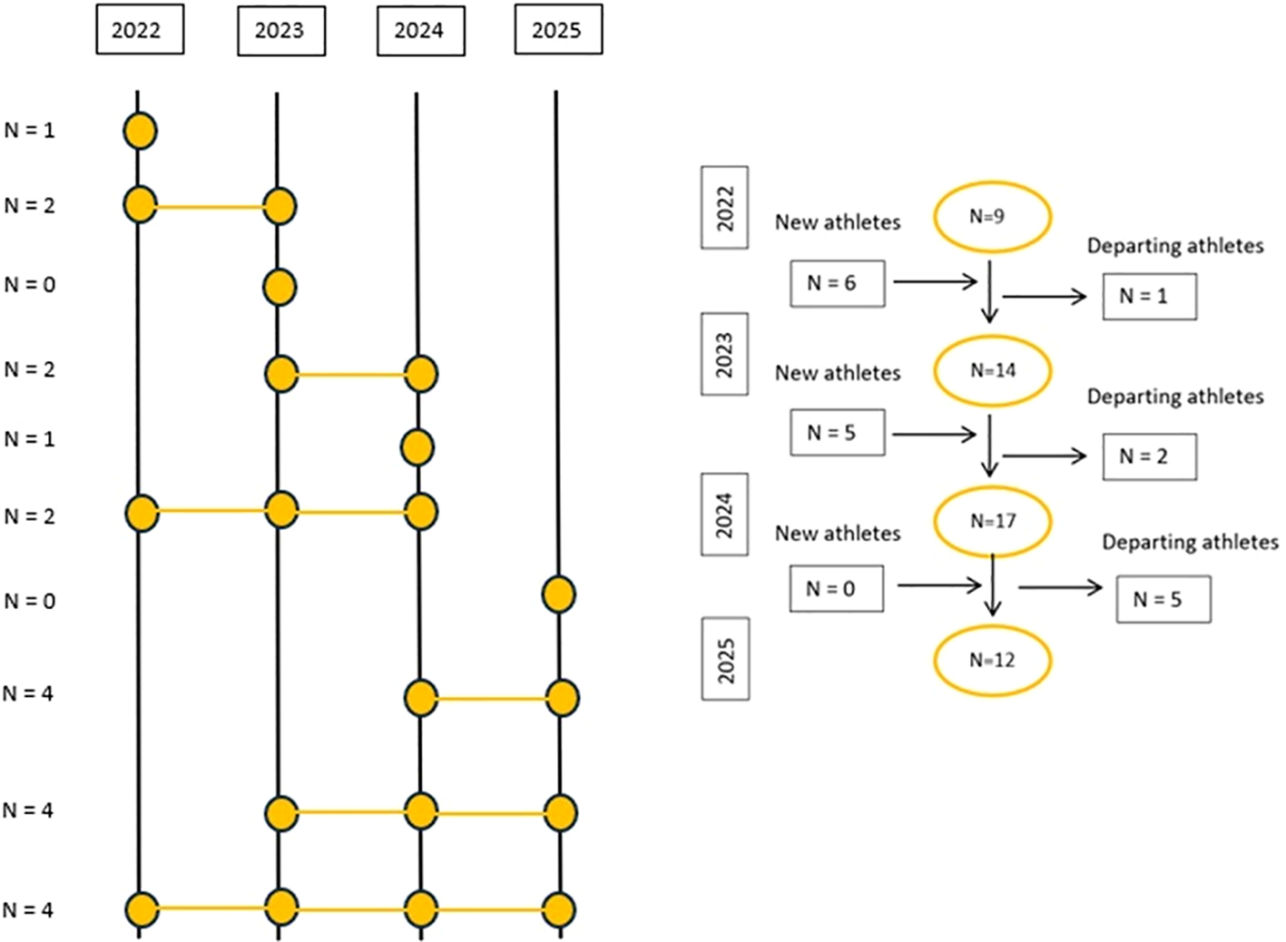
Number of athletes in the training group across each season and number of new and departing athletes each season.

A longitudinal and descriptive study was conducted. The information presented in this article corresponds to the athletes who were part of the training group during the 2022, 2023, 2024 and 2025 seasons. A total of 20 triathletes were monitored across the four consecutive years of the study (**Figure 1**). All athletes belonged to the same training group, followed the same training methodology and were led by the same coaches. The number of participating athletes was 9, 14, 17, and 12 for 2022, 2023, 2024 and 2025, respectively. Four male athletes were present throughout all 4 consecutive years of the program. Data from all four seasons were included in the analyses, and the values presented represent averaged results across the monitoring period. The unit of analysis was the individual measurement occasion within each season, while each season was conceptually treated as an independent adaptation cycle within the overall longitudinal monitoring framework. This approach is consistent with the nature of high-performance training, where each season represents a distinct physiological process. Consequently, athletes’ responses to training stimuli are expected to evolve over time, reflecting progressive adaptations and increased tolerance to training load. As performance level improves, greater training stimuli are required to elicit further adaptations, leading to non-stationary intra-individual responses across seasons. Therefore, treating each season as an independent adaptation cycle allows for a more ecologically valid interpretation of the data within the context of elite endurance training.

During these seasons, individual training load data were collected during the general and specific preparatory period. A traditional periodization plan was followed in this part of the season. This traditional periodization plan was characterized by clearly defined preparatory phases, including a general preparatory period focused on developing aerobic capacity and general physical qualities, followed by a specific preparatory period with progressively increased intensity and greater specificity to competitive demands.

The general preparatory period lasted 12 ± 1 weeks on average. The specific preparatory period lasted 9 ± 1 weeks on average. In the 2022 season, the general preparatory period lasted 11 weeks and the specific preparatory period 10 weeks. In 2023, the durations were 12 weeks for the general preparatory period and 9 weeks for the specific preparatory period. In 2024, the general preparatory period comprised 13 weeks and the specific preparatory period 9 weeks, while in 2025 the general preparatory period lasted 12 weeks and the specific preparatory period 10 weeks. Each mesocycle was composed of a minimum of 3 microcycles and a maximum of 5 microcycles. Each microcycle was equivalent to 1 week.

The training schedule was designed by the head coach (R.C). Training was planned according to athletes’ competitive objectives. **Table 1** shows the generalized goals per mesocycle during this period. At the beginning of the season, athletes were categorized according to their level of competitive performance and their ability to assimilate the training load (McKay et al., 2022). The training doses and volume varied depending on the performance characteristics of the triathlete (Cardona et al., 2019), but the training program was the same for all participants during this part of the season in terms of intensity distribution and the percentage of training load allocated to each segment. In addition, acute training load was categorized and systematically compared across microcycles.

**Table 1:** Order of priority per goal of the general and specific preparatory period.

Period	Mesocycle	Duration (Weeks)	TL hardness	Basic physical conditioning	Ventilatory threshold 1	Ventilatory threshold 2	Maximal aerobic speed/power	Basic strength
General preparatory period	1	5	Low	****	**	*	–	****
	2	4	Low to moderate	***	****	**	*	****
	3	4	Moderate to severe	*	****	***	**	***
Specific preparatory period	4	3	Severe	–	***	****	***	**
	5	3	Severe	–	**	****	****	*
	6	3	Moderate to severe	–	**	****	****	*

*Degree of importance of the topic from 1 to 4, with a 4-star rating being the most important, TL, Training load.

Capillary blood samples were collected at multiple time points to monitor urea and CK concentrations. Baseline measurements were obtained during the week preceding the start of the season, following a three-week period without training. Subsequent samples were collected on the last day of the penultimate microcycle of each mesocycle throughout the monitoring period. This penultimate microcycle corresponded to the highest training load within the mesocycle and served as a measure of the level of physiological stress induced by training. Values were not measured in the first mesocycle as it was considered that the physical conditioning of the participant would not be optimal and could alter the results (Haller et al., 2023). Therefore, the first assessment was postponed until athletes had completed an initial period that allowed them to adapt to the training load. The study did not continue into the competitive period, as travel commitments and the heterogeneity of competition schedules made it impossible to maintain the systematic monitoring of the aforementioned variables.

**Figure 2:**
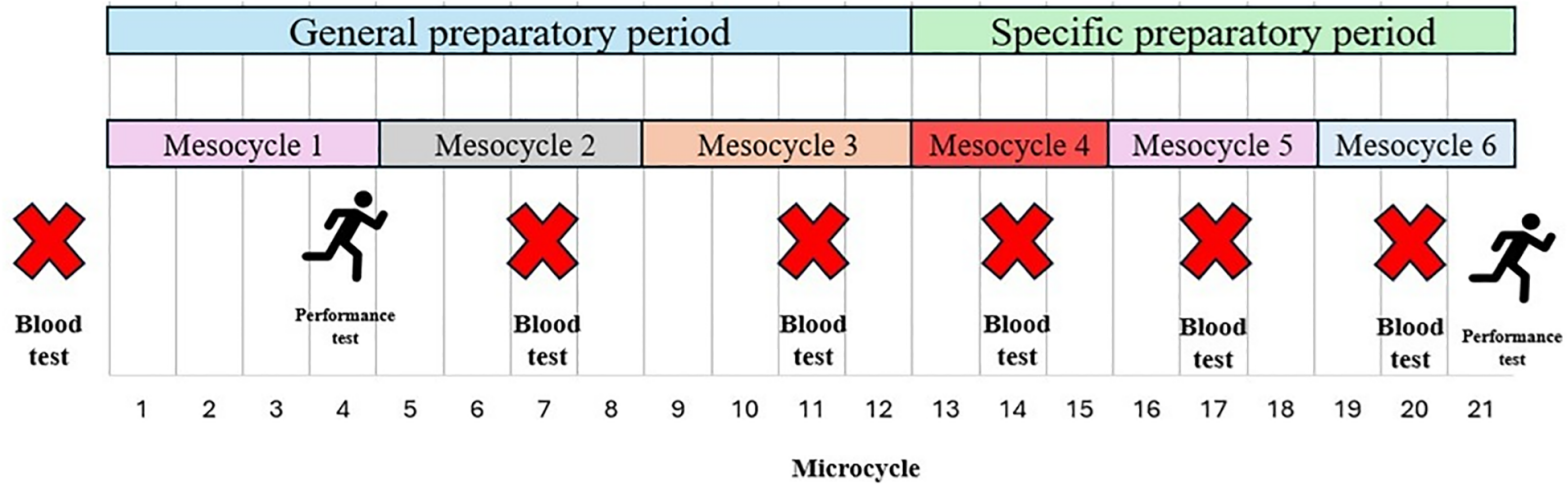
Example of test and training schedule.

Additionally, performance tests in cycling, were conducted at the beginning of the general preparatory period (Test 1, during week 4) and at the end of the specific preparatory period (Test 2) to evaluate changes in physiological performance. **Figure 2** shows an example of periodization and testing Schedule for the study.

At the beginning of the season, all participants underwent a medical examination to confirm their physical suitability for high-intensity training. Informed consent was obtained from all subjects prior to data collection, allowing their information to be used for research purposes. The study protocol received approval from the Ethics Committee of the University (A- 2017-04–11 expedient), and all procedures adhered to the principles outlined in the Declaration of Helsinki.

### 2.2 Participants

According to McKay et al.’s (2022), 2 athletes were identified as tier 5 (World class level) athletes, 9 as tier 4 (international-level) athletes, and 9 as tier 3 (national-level) athletes. Therefore, 11 were considered as international triathletes and 9 as national level triathletes. International level participants were professional athletes who dedicated themselves exclusively to triathlon training and competition. The tier 3, national-level, athletes were university students with dual academic and sporting careers. The study subjects included 2 triathletes with at least 1 podium finish in the World Triathlon Series, 3 triathletes with at least 1 podium finish in the world triathlon cup and 5 national champions. The two world-class athletes had an average age of 25.5 ± 4.9 years, an average height of 175.0 ± 8.5 cm, an average body mass of 61.6 ± 1.6 kg, and an average running V̇O_2_max of 83.7 ± 0.4 mL·kg^-^¹·min^-^¹. The nine international-level athletes had an average age of 22.3 ± 1.6 years, an average height of 171.4 ± 4.6 cm, an average body mass of 60.8 ± 4.2 kg, and an average running V̇O_2_max of 82.8 ± 1.4 mL·kg^-^¹·min^-^¹. The nine national-level athletes had an average age of 21.2 ± 1.3 years, an average height of 175.5 ± 5.7 cm, an average body mass of 66.9 ± 6.7 kg, and an average running V̇O_2_max of 81.5 ± 2.6 mL·kg^-^¹·min^-^¹.

### 2.3 Performance tests

Cycling tests were conducted to assess performance improvements over time. For cycling, an incremental ramp test to volitional exhaustion was performed using an athlete’s own bicycle mounted on a portable electromagnetically braked trainer (Wahoo^®^ KICKR Power Trainer, Atlanta, GA, USA), starting at 100 W and increasing by 5 W every 12 seconds (Muñoz et al., 2014). Power increments were manually controlled by the researcher via the Wahoo App^®^ (Atlanta, GA, USA).

During cycling tests, a portable gas-exchange analyzer (Cosmed^®^ K5, Rome, Italy) was used to establish intensity zones based on ventilatory thresholds (VT) and Maximal oxygen uptake (V̇O_2_max). Measured variables included oxygen uptake (VO_2_), pulmonary ventilation (VE), ventilatory equivalents for oxygen (VE/VO_2_) and carbon dioxide (VE/VCO_2_), and end-tidal partial pressures of oxygen (PETO_2_) and carbon dioxide (PETCO_2_). V̇O_2_max was defined as the highest average VO_2_ over any continuous 1-minute period showing a plateau (Le Meur et al., 2009). Ventilatory thresholds were determined using the Davis criteria (Davis, 1985): VT1 was identified as an increase in VE/VO_2_ and PETO_2_ without a rise in VE/VCO_2_, while VT2 was defined by increases in VE/VO_2_ and VE/VCO_2_ and a decrease in PETCO_2_. VT1 and VT2 [and the corresponding relative power (W/kg) or speed (S)] were independently identified by two experienced observers following a visual inspection of the gas-exchange data. Subsequently, both observers conducted a joint review of the ventilatory graphs to reach a consensus on threshold identification. For the final analysis, the mean value derived from both observations was used. Heart rate was continuously monitored throughout the tests using radio telemetry (Polar Electro^®^ Verity Sense, Kempele, Finland).

### 2.4 Training load monitoring

To quantify training load and control session intensity, a triathlon-specific methodology was employed: Objective Load Equivalents (ECOS, from the Spanish acronym) (Cejuela and Esteve-Lanao, 2011). The ECOS model was selected for training load quantification due to its specific suitability for triathlon, as it accounts for the unique characteristics and demands of this multisport discipline. Unlike more generic models, ECOS integrates the type of training performed across swimming, cycling, and running, providing a more sport-specific assessment of training load. Furthermore, this model has been previously applied in studies involving elite triathletes, supporting its relevance and practical applicability in high-performance contexts (Arévalo-Chico et al., 2024, 2025; Cejuela and Selles-Perez, 2023).

Eight training intensity zones were established based on individual performance test previously made. These zones integrated both internal load indicators (e.g., heart rate) and external load measures (e.g., speed or power output) and were aligned with a 1–10 rating of perceived exertion (RPE) scale.

Training load was calculated by multiplying the time (in minutes) spent in each intensity zone (1 to 8) by a corresponding weighting factor (ranging from 1 to 50). This resulting value was further adjusted according to the discipline-specific coefficient: 1.0 for running, 0.75 for swimming, and 0.5 for cycling (Cejuela and Esteve-Lanao, 2011). Heart rate and RPE were primarily used to regulate low-intensity sessions (zones 1–2), whereas speed or power output were used for moderate- to high-intensity training (zones 3–8).

In addition, Subjective Load Equivalents (ECS, by their Spanish acronym) were recorded. This subjective model required athletes to rate each training session on a 0–5 scale based on their perceived effort (Cejuela and Esteve-Lanao, 2011). Both objective (ECOS) and subjective (ECS) models were used throughout the study to monitor training load.

Chronic training load was calculated as the sum of ECOS accumulated over the preceding 21 days, reflecting long-term training exposure. Acute training load was defined as the sum of ECOS and ECS accumulated over the previous 7 days, providing an estimate of short-term training stress (Cardona et al., 2019).

The ECOS/ECS ratio (ECOS/ECS_ratio_) was subsequently calculated using acute values, combining objective load quantification with the athlete’s subjective perception of effort. This ratio provides an index of the relationship between the prescribed training dose and the perceived effort, where higher values may reflect a lower subjective strain relative to the objective load and potentially more favorable adaptation, whereas lower values may indicate a disproportionate perceived effort and a possible state of excessive physiological stress. All calculations were performed using dedicated software (All in Your Mind Training 143 system, Mexico).

Acute training load was categorized as low, moderate, or severe based on thresholds previously reported in national- and international-level triathletes (Arévalo-Chico et al., 2025). Severe load was defined as acute ECOS values exceeding the reference mean by more than 30%, moderate load as values within the reference range, and low load as values at least 15% below it. Specifically, severe load corresponded to ECOS values >1500 units for international-level athletes and >1125 units for national-level athletes; moderate load ranged between 980–1500 and 740–1125 units, respectively; and low load was defined as <980 units for international athletes and <750 units for national athletes.

### 2.5 Biochemical measures

Capillary blood samples were taken from the subjects using equipment specialized in the measurement of urea and CK under these conditions (Fujifilm dri-chem nx600, Fujifilm Corporation, Saitama, Japan). All blood samples were taken in a quiet laboratory room maintained at a constant temperature of 20 – 22C. All samples were taken in a fasted state and at standard times between 8:00 and 10:00 a.m. to avoid variations in circadian rhythms. All subjects were asked to avoid consuming products containing caffeine and alcohol during the 24 h prior to blood collection. All participants followed a nutritional plan designed by a qualified sports nutritionist. Adherence to the nutritional protocol was monitored through monthly follow-up meetings conducted throughout the study period. During these meetings, athletes reviewed their dietary practices and confirmed compliance with the prescribed guidelines. No major deviations from the recommended diet were reported during the monitoring period. One 300 μL sample of blood was collected from the fingertip using a lancet and placing it in a tube with lithium heparin (Microvette CB 300-LH, Sarstedt, Germany). Once the sample was extracted, it was left to stand for 60 minutes at a constant temperature of 20 – 22C. The sample was then centrifuged for 10 min at 4000 g. Urea and CK levels were then calculated from the blood serum sample.

### 2.6 Statistical analysis

Non-parametric statistical tests were applied to analyze differences in training load, urea, and CK across the different study periods. Specifically, the Wilcoxon test was used to compare differences between related samples across consecutive measurement time points and between performance at test 1 and test 2. To examine overall differences in biomarker concentrations across all measurement time points, a Friedman test for multiple related samples was performed. In addition, the Mann–Whitney U test was used to assess potential differences between performance-level groups (e.g., national- vs. international-level athletes) in training load, urea, CK values, and performance changes between Test 1 and Test 2.

Effect size was measured using the Cohen’s d (Cohen, 1992). Cohen’s d was interpreted as follows: trivial: <0.25, small: |0.25| - |0.5|, moderate |0.5| - |1|, large: |>1| (Rhea, 2004). In addition, 95% confidence intervals and mean differences expressed as percentages were calculated. Spearmans’ bivariate correlation coefficient was used to determine the inter-relationships between training load, changes in performance and urea and CK values.

To obtain a correlation coefficient of at least 0.600 with a significance level of 0.05 and a statistical power of 80%, a minimum of 19 participants were needed. The statistical software Epidat 4.0 (Epidat, DXSP, Spain) was used to perform the sample size estimation. The statistical software used for the statistical analysis was Statistical Package for Social Sciences (SPSS) 22.0 (SPSS Inc., Chicago, IL, USA). Differences were accepted as significant as long as p < 0.05.

## 3 Results

**Figure 3:**
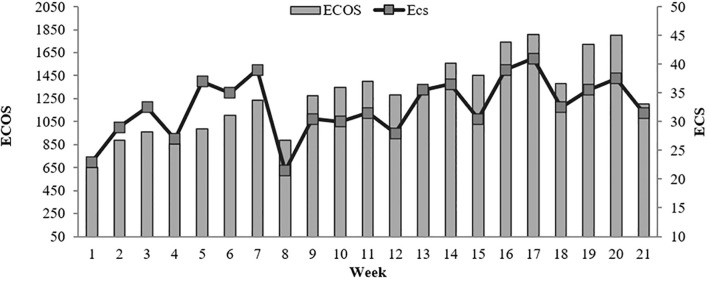
Example of evolution of training load during the monitoring period. ECS, Subjective training load Equivalent; ECOS, Objective training load Equivalent.

An example of training load progression during the monitoring period is shown in **Figure 3**, illustrating the variations in workload across the microcycles.

**Table 2** presents the values of blood urea and CK measured at baseline and during the training periods in both national- and international-level athletes. The table shows the differences between baseline and in-season measurements across the general preparatory and specific preparatory periods. Additionally, training load values recorded during each training period are reported separately for national and international athletes.

**Table 2:** Hematological and training variables in national- and international-level triathletes during the recording period.

Measure	Baseline (1 test)			General preparatory period (average of 2 test)			Specific preparatory period (average of 3 test)			Friedman test for multiple related samples (*p*)		
	Int	Nat	CI 95%	Int	Nat	CI 95%	Int	Nat	CI 95%	Int	Nat	Avg
Haematological Variables												
Urea (mg · L ^–1^)	36.7 ± 12	34.1 ± 9.9	-12.8 to 6.4	47.1 ± 14†	46 ± 13.2†	-14.2 to 7.2	51.9 ± 11.9	47.5 ± 16.2	-11.7 to 1.7	0.20	0.39	0.32
Δ Urea (%)				28.3 ± 26.2	34.8 ± 25.3	-68.7 to 60.5	41.4 ± 23.3†	39.3 ± 14.9	-25.9 to 24.7	0.19	0.31	0.27
CK (U·L ^–1^)	128 ± 106	153 ± 98	-201.5 to 167.9	226 ± 195†	350 ± 204†	-39.7 to 300.6	195 ± 183	242 ± 197	-37.6 to 135.7	0.14	0.22	0.31
Δ CK (%)				76.6 ± 73.1	128 ± 110.1	-79.5 to 391.5	52.3 ± 64.2†	58.2 ± 64.3†	-52.8 to 82	0.13	0.09	0.22
Training Variables												
AATL (Total ECOS)				1285 ± 240	1203 ± 372	-266.2 to 287.4	1318 ± 330*	1251 ± 358*	-245.5 to -1	0.20	0.21	0.23
ACTL (Total ECOS)				4010 ± 904	3848 ± 1420	-1077 to 303	4115 ± 1094*	3734 ± 804*	-232.8 to -30.6	0.14	0.19	0.19
AATL (Total ECS)				34.2 ± 13.6	30.4 ± 11.5	-21.8 to 7.6	39.2 ± 11.9*	30.2 ± 9.8*	-16.7 to -4.9	0.25	0.63	0.32
ECOS/ECS_ratio_ (U.A)				37.6 ± 9.2	39.6 ± 12.1	-6.4 to 19.6	33.6 ± 9.9*†	41.1 ± 12.2*	2.2 to 13.8	0.26	0.29	0.21

†Difference with the previous period, assessed using Wilcoxon test; *Difference with performance group, assessed using Mann Whitney U; Δ, Change; CK, Creatine kinase; AATL, Average acute training load; ACTL, Average chronic training load; Int, International level athlete; Nat, National Level Athlete. ECs, Subjective training load Equivalent; ECOs, Objective training load Equivalent; CI 95%, Confidence interval 95% between performance group; Avg, Average.

It is important to highlight the high variability observed in biomarker data, as reflected by the standard deviations across all parameters. However, it can be observed that urea and CK values were statistically higher during the general preparatory period compared to baseline data. During the specific preparatory period, an increase in urea values was also observed in international-level athletes, whereas no such change occurred in national-level athletes. All groups showed a decrease in CK levels during the specific preparatory period. Similarly, the absence of statistically significant differences in the Friedman test across measurements at different time points demonstrates that the high inter-individual variability observed in urea and CK responses limits the ability to establish direct cause–effect relationships based on isolated measurements. During the general preparatory period, international-level athletes showed higher training-load values (measured with ECOS and ECS) although these differences were not statistically significant. With regard to the ECOS/ECS_ratio_, both international- and national-level athletes showed similar values during the general preparatory period. However, during the specific preparatory period, international-level athletes exhibited a decrease in this ratio compared with the previous period, and their values were significantly lower than those of national-level athletes.

**Figure 4:**
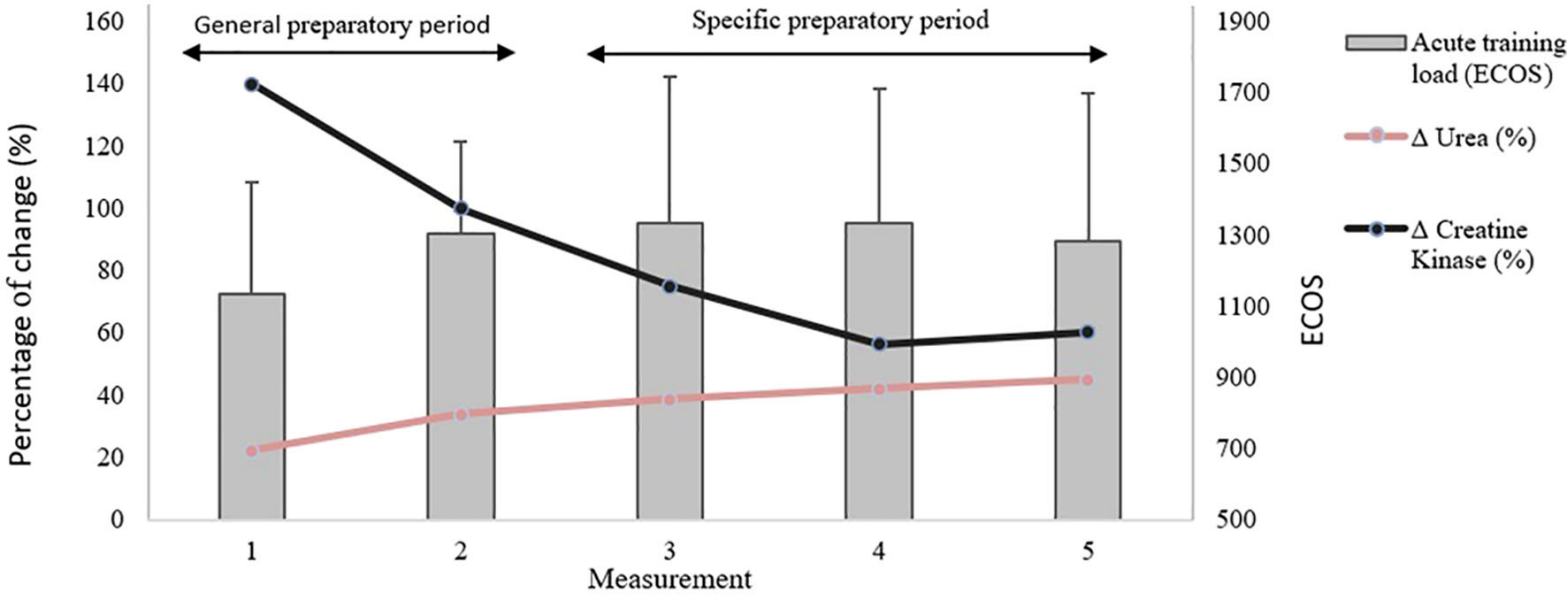
Evolution of training load and percentage changes in urea and creatine kinase relative to baseline. ECOS, Objective training load Equivalent.

**Figure 5:**
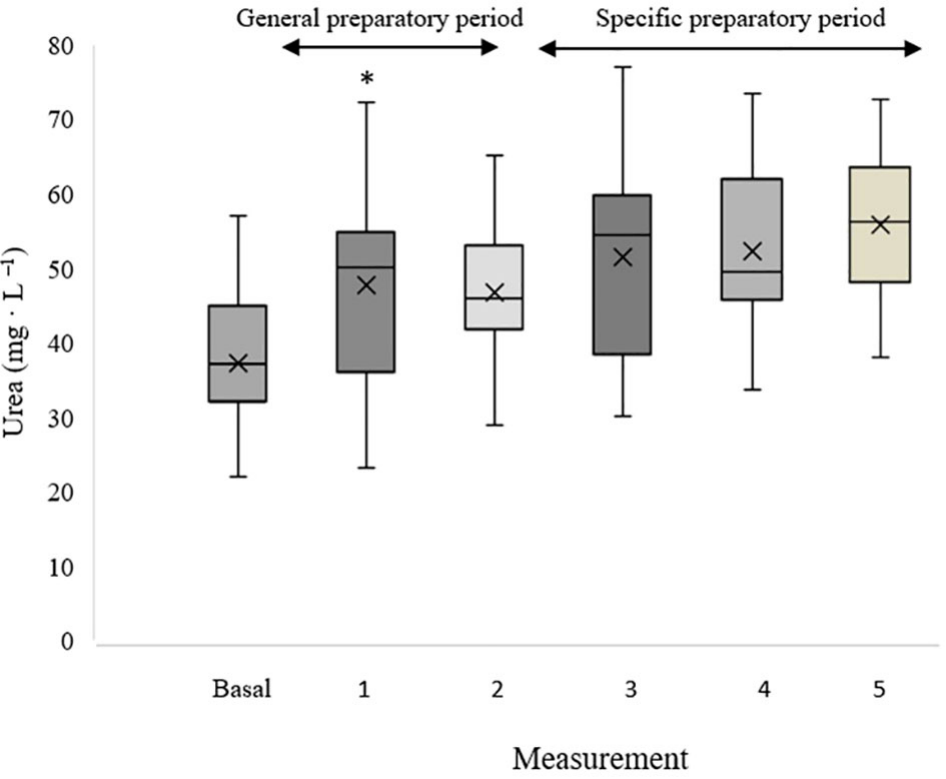
Absolute urea values at baseline and during the recording period. *Statistic difference with the previous period (p<0.05).

**Figure 6:**
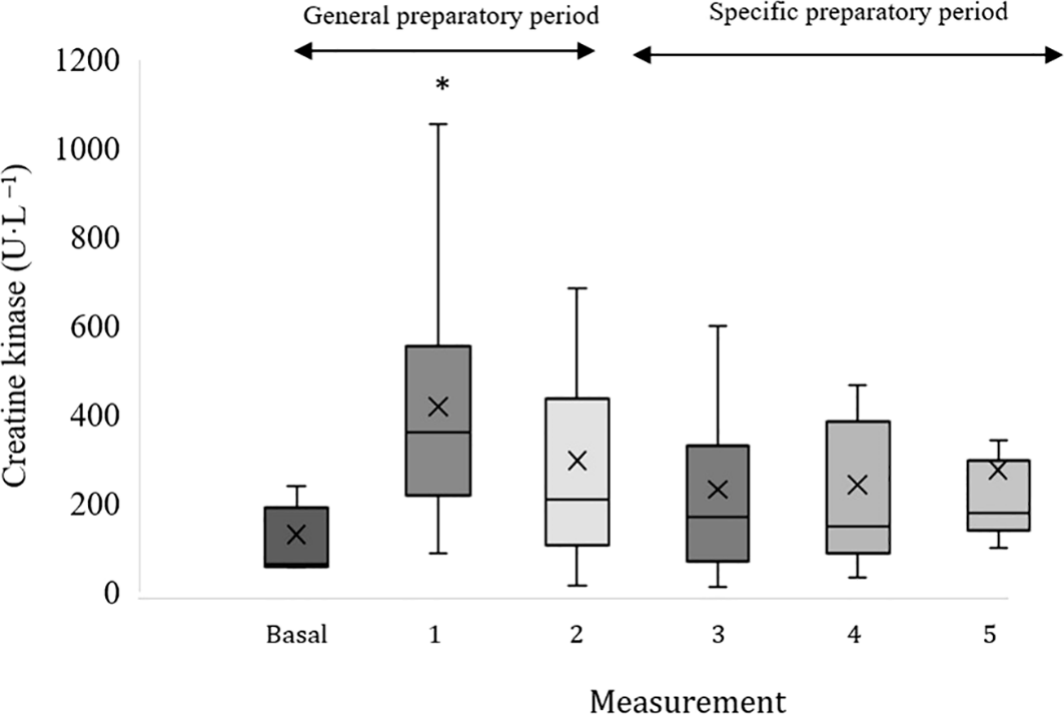
Absolute creatine kinase values at baseline and during the recording period. *Statistic difference with the previous period (p<0.05).

**Figure 4** shows the evolution of the changes (as percentage of change) in blood urea and CK levels from baseline throughout the competitive season, alongside the corresponding training load values. **Figures 5** and **6** shows the absolute values of blood urea and CK, respectively across all measurement points during the monitoring period. In these figures, an upward trend in urea values can be observed throughout the monitoring period, as well as a decline followed by stabilization in CK levels. The high inter-individual variability in blood biomarker values remains evident.

**Table 3** shows the comparison between baseline urea levels and those measured during period of severe, moderate, and low acute training loads. The data show statistically significant differences between baseline values and those recorded during severe and moderate acute-load periods, but not during low acute-load periods. Furthermore, the effect size was larger (d=2.1) in severe-load microcycles than in moderate-load microcycles (d=1.21). The effect size was trivial during periods of low acute training load (d=0.13).

**Table 3:** Comparison of urea values as a function of acute training load hardness and differences with basal values.

Acute training load hardness	Percentage of measurements	Urea value average (mg · L ^–1^) and standard deviation	ES (Cohen’s d)	p	CI 95%	PC basal (%)
Severe	42.4	51.2 ± 12.7	2.10	<0.001	14.7 to 22	43.5
Moderate	42.2	48.3 ± 11.3	1.21	<0.001	7.0 to 13.9	35.3
Low	15.4	41.1 ± 13.7	0.13	0.142	-8.4 to 1.3	8.1

ES, Effect size; p, statistical significance; CI 95%, confidence interval 95%; PC, Percentage of change; %, percentage.

Regarding performance-related physiological variables, **Table 4** presents the data from performance Tests 1 and 2, along with the percentage difference between the two tests. International-level athletes achieved better results in both tests and showed greater performance improvements from Test 1 to Test 2. Statistically significant differences were observed between Test 1 and Test 2 for all physiological performance variables, except for VO_2_max in the national-level group. However, no statistically significant differences were found between groups in the magnitude of performance improvements.

**Table 4:** Performance test results and between-group improvement data.

	Test 1		Test 2		Improvement							
					Int				Nat			
	Int. Avg	Nat. Avg	Int. Avg	Nat. Avg	PC (%)	p	ES	CI 95%	PC (%)	p	ES	CI 95%
V̇O_2_max (mL/kg/min)	73.7 ± 4.1	72.5 ± 7.5	81.7 ± 8.4*	78.0 ± 8.2	10.2 ± 4.8	0.03	-0.91	-16.6 to -1.42	7.4 ± 6.0	0.07	-.81	-14.5 to 3.5
Relative power at V̇O_2_max (W/kg)	6.3 ± 0.5	6.1 ± 0.7	6.7 ± 0.5*	6.4 ± 0.6	6.6 ± 1.6	0.04	-0.90	-0.66 to -0.24	4.5 ± 6.3	0.14	-0.66	-0.8 to 0.2
Relative power at VT_2_ (W/kg)	4.8 ± 0.5	4.6 ± 0.3	5.1 ± 0.6*	4.8 ± 0.3*	7.8 ± 1.2	0.04	-0.91	-0.58 to -0.27	4.1 ± 2.5	0.04	-0.91	-0.35 to -0.47
Relative power at VT_1_ (W/kg)	3.4 ± 0.4	3.2 ± 0.2	3.8 ± 0.2*	3.4 ± 0.2*	9.9 ± 7.1	0.03	-0.93	-0.80 to - 0.04	6.2 ± 3.6	0.04	-0.9	-0.38 to -0.05

*, Statistic difference with test 1, assessed using Wilcoxon test; Int. Avg, Average value of international athletes; Nat. Avg, Average value of national athletes; PC, Percentage of change; p, statistical significance; ES, Effect size, assessed using Cohen’s d; CI 95%, Confidence interval 95%.

Focusing on the correlation analysis, a positive association was found between the average increase in urea values during the recording period relative to baseline and the average ECS values recorded (*ϱ* = 0.67; p < 0.05). Additionally, a statistically significant negative correlation was observed between this average urea increase and the mean ECOS/ECS_ratio_ (*ϱ* = -0.55; p < 0.05). It is important to highlight that no statistically significant correlations were found between ECOS values and any of the variables monitored. A statistically significant negative correlation was also found between improvements in relative power at V̇O_2_max and VT2 and the average increase in urea ((*ϱ* = -0.65; p < 0.05) (*ϱ* = -0.68; p < 0.05) respectively). No statistically significant correlations were found between CK values and any of the variables monitored in the present study.

## 4 Discussion

The purpose of this study was to examine the relationship between performance changes, training-load doses, and fatigue-related biomarkers—specifically blood urea and CK—across the general and specific preparatory periods in elite triathletes, using a training-load model that integrates both objective and subjective metrics. Additionally, the study aimed to explore potential differences in the dose–response relationship between athletes of varying competitive levels, providing insight into how training adaptations and evolution on performance may differ based on performance status.

The relationship between training load and blood urea concentrations appears to be well established in cyclic endurance sports (Greenham et al., 2018). In these modalities, increases in training intensity and volume are commonly accompanied by elevated urea levels, reflecting increased protein turnover and metabolic stress (López-Chicharro and Fernández-Vaquero, 2022). This trend supports the use of urea as a potential indicator of training strain in endurance disciplines, although its interpretation should consider individual variability and external factors such as hydration status and nutritional intake (Djaoui et al., 2017).

In the present study, a relationship between training load and elevated urea levels can be observed when comparing baseline values to those recorded during periods of severe, moderate, and low acute training load. The largest effect sizes were found during phases of severe training load, followed by moderate periods, while no significant differences were observed during low-load phases. These results reflect more pronounced changes in urea compared to other studies (Greenham et al., 2018). This could be attributed to the high training volumes of the triathletes involved (Cejuela and Selles-Perez, 2023) and the fact that baseline urea was measured after a three-week recovery phase between seasons.

Additionally, the correlation analysis shows that the triathletes who recorded higher ECS values experienced a greater increase in mean urea levels throughout the monitoring period. However, no meaningful relationships were found between ECOS values and urea concentrations. This outcome may reflect the fact that objective training-load metrics do not always capture the true magnitude of physiological strain imposed by training. In contrast, variables related to subjective perception may offer a more sensitive representation of the athlete’s internal response, integrating factors such as fatigue, motivation, and accumulated stress that external metrics alone cannot fully account for (Haddad et al., 2017). This limitation may be even more evident when comparing heterogeneous groups of athletes, where individual differences in training history and tolerance to load can influence biomarker responses independently of the external workload performed. Likewise, athletes who displayed lower ECOS/ECS_ratio_ showed elevated urea values, suggesting a potential relationship between excessive physiological stress and an imbalance between the prescribed workload and the perceived effort. Higher ECOS/ECS_ratio_ values may be associated with more favorable adaptations to the training dose, as they indicate a lower perceived exertion relative to the objectively prescribed load, potentially reflecting a greater tolerance to training stress. This result underscores the value of combining objective and subjective methods of training-load monitoring, as their integration can provide critical insights into an athlete’s fatigue status and overall preparedness. By leveraging both perspectives, practitioners can obtain a more holistic understanding of the athlete’s response to training and make more informed decisions to optimize performance and recovery (Halson, 2014).

A clear trend toward increasing urea levels was observed over the course of the season. Furthermore, these values exceeded in all cases the normal values in the general population. This pattern may be explained by the progressive rise in training demands. The observed increases in urea concentrations are consistent with findings from other studies conducted in endurance athletes, both during short-term training blocks (Hecksteden et al., 2016) and over extended training periods (Zapico et al., 2007). This suggests a progressive increase in physiological stress as the season advances, particularly during phases of intensified training load. Further studies are needed to analyze changes in urea levels during competition periods as well. However, it is likely that additional confounding variables—such as travel related to competitions—could influence the results during this period of the season.

No significant relationships were found between CK levels and training load in this study. CK is an enzyme commonly used as a biomarker of muscle damage, as it is released into the bloodstream following structural disruption of muscle fibers (López-Chicharro and Fernández-Vaquero, 2022). However, in the context of triathlon—an endurance sport characterized by predominantly concentric, cyclical movements and minimal impact or contact—the extent of exercise-induced muscle damage may be lower than in sports involving eccentric loading or physical contact (Meyer and Meister, 2011). This could explain the lack of association between CK levels and training load, suggesting that CK may be less sensitive for monitoring training stress in triathletes compared to other populations.

However, a clear reduction in CK levels was observed throughout the training period. This trend may indicate that greater muscle damage occurs at the beginning of the training cycle, gradually decreasing as the musculature adapts to the imposed load (López-Chicharro and Fernández-Vaquero, 2022). Additionally, the emphasis on strength training during the early phases of the season—particularly in the general preparatory period—could partially explain the higher CK values recorded at the start. Given this pattern, monitoring CK concentrations may be useful at the beginning of the season to help prevent excessive muscle damage that could increase the risk of injury in elite triathletes (Arévalo-Chico et al., 2024). Further research is needed to explore this issue in greater depth and confirm these observations.

Despite the trends observed in biomarker behavior throughout the monitoring period, the substantial inter-individual variability evident across measurements suggests that, at an acute level, establishing direct cause-and-effect relationships between training load and physiological responses is not appropriate. Factors such as sleep quality, nutrition, psychological state, or personal circumstances can meaningfully modulate urea, CK, and other biomarker responses, either masking or amplifying the effects attributable to external or internal load (Noone et al., 2024; Biljak et al., 2025).

In addition, the concept of delayed training effects should be considered when interpreting the present findings. Physiological adaptations and fatigue-related biomarker responses do not always occur immediately after the application of a given training stimulus but may manifest after a temporal delay depending on the magnitude, type, and accumulation of training stress. Therefore, inter-individual differences in recovery kinetics and adaptation timing may have contributed to the heterogeneous biomarker responses observed. This reinforces that single measurements of urea or CK cannot be used to identify excessive fatigue. Their utility emerges only through repeated, longitudinal tracking, especially when elevated values are sustained over multiple consecutive assessments. Therefore, an individualized, holistic and longitudinal monitoring approach is essential. Further work is needed to develop athlete-specific frameworks that more accurately link biomarker fluctuations with the corresponding training-load demands and responses.

In this study, international-level triathletes recorded higher training loads compared to national-level athletes, although statistically significant differences were only observed during the specific preparatory period, when overall training load was highest. Despite these differences, no significant differences were found in CK or urea levels between the two groups at any time point. This may suggest that, although training doses differ, the physiological responses remain similar, possibly because neither group exceeded their maximum tolerable load threshold. Furthermore, in periods where training loads did not differ significantly between groups, no significant differences were observed in biomarker levels either. This could indicate that part of the ability to tolerate and adapt to training load is independent of purely physiological stress responses and may be influenced by other behavioral or psychological factors, such as motivation toward training and commitment to competitive goals (Issurin, 2017).

A negative relationship was found between improvements in VT2 and V̇O_2_max power output and the increase in blood urea levels. This may indicate that excessive physiological fatigue can limit performance gains in endurance athletes. Although the large interindividual variability makes urea unsuitable as a single marker to adjust training loads, monitoring blood urea during preparatory periods may still help identify states of excessive fatigue that hinder performance improvement. These findings can be interpreted within a dose–response framework, where the relationship between training load and physiological response is not linear and is modulated by individual tolerance. Similarly, the observed patterns may be partially explained by the fitness–fatigue model, in which excessive training stress may transiently impair performance despite ongoing adaptations (Morton et al., 1990). Additionally, conducting longitudinal and ecologically valid studies in elite athletes remains particularly challenging due to constraints related to training schedules, competition demands, and limited accessibility (Sandbakk et al., 2023). Therefore, more research is needed to develop methods for setting individualized cut-off values that allow practitioners to detect abnormal increases during training (Schuth et al., 2023).

From a practical perspective, the combined use of objective (ECOS) and subjective (ECS) training load measures, together with the ECOS/ECS_ratio_, provides coaches with a simple and actionable tool to monitor the balance between the prescribed training dose and the athlete’s internal response. Importantly, the finding that subjective load shows stronger associations with physiological responses than objective load highlights the relevance of incorporating athletes’ perceptual feedback as a key element in the monitoring process. This approach allows practitioners to detect potential mismatches between external load and perceived effort, facilitating early identification of excessive fatigue or maladaptive responses. Furthermore, the categorization of acute training load into low (<980 units for international athletes and <750 units for national athletes), moderate (between 980–1500 and 740–1125 units for international and national athletes respectively), and severe (>1500 units for international-level athletes and >1125 units for national-level athletes) enhances decision-making in day-to-day training adjustments, making this model particularly applicable in real-world high-performance environments where rapid and minimally invasive monitoring is required.

### 4.1 Limitations

This study provides valuable insight into the physiological responses to training in high-level endurance athletes and supports the use of mixed training-load quantification models. However, several limitations must be acknowledged. Monitoring did not cover the entire season, and long intervals without blood sampling may have increased biomarker variability and reduced the ability to detect consistent trends. Only one training-load model was used, preventing comparisons with other quantification approaches. In addition, relevant biomarkers linked to fatigue and adaptation—such as the testosterone–cortisol ratio or oxidative stress indicators—were not assessed. The inclusion of these markers could have provided a more comprehensive understanding of the physiological stress imposed by training, as well as the balance between anabolic and catabolic processes and the athletes’ recovery status.

On the other hand, the potential differential effects of the three triathlon disciplines (swimming, cycling, and running) on biomarker responses were not specifically addressed. Each discipline imposes distinct mechanical and physiological demands, which may lead to different patterns of muscle damage and metabolic stress, potentially influencing biomarkers such as urea and CK.

Another limitation of this study is that physiological performance was assessed only through cycling testing, despite triathlon being a multidisciplinary sport. Although adaptations may differ across swimming, cycling, and running, cycling was the only discipline for which performance testing was conducted consistently across all participants and seasons. While this approach ensured methodological consistency, future studies should incorporate discipline-specific assessments to provide a more comprehensive evaluation of training adaptations in triathletes.

An additional consideration of this study is the inclusion of only male athletes from a single training group trained by the same coach. Although this approach provided methodological consistency in training prescription and monitoring, it may limit the generalizability of the findings to female athletes, other training environments, or coaching contexts. Future research should include more diverse samples and multiple training groups to confirm the applicability of these results across different populations and settings.

These omissions limit the scope of the physiological interpretation. It is also important to consider external factors that may influence adaptive responses, including individual characteristics, hydration status, sleep, recovery and behavioral factors (Pickering and Kiely, 2017). Future studies should incorporate more comprehensive biomarker panels and adopt specific modelling approaches, such as the fitness–fatigue (Morton et al., 1990), to better disentangle the effects of acute and chronic loads on physiological markers like urea. Additionally, future studies should consider the application of mixed-effects models to better account for individual variability and longitudinal responses.

## 5 Conclusions

This study suggest that higher training loads are associated with increases in urea concentrations in elite triathletes, particularly under severe and moderate acute load conditions, whereas CK does not exhibit a meaningful association with endurance training stress. Athletes who accumulated greater subjective training-load values based on perceived exertion showed proportionally larger increases in urea. In contrast, lower ECOS/ECS_ratios_ were associated with greater increases in urea, indicating that the interaction between objectively monitored load and perceived exertion may reflect physiological strain with notable fidelity.

The absence of differences in urea fluctuations between national- and international-level triathletes, despite phases with comparable training loads, suggests that adaptive capacity is influenced by factors beyond workload metrics alone.

The negative associations between performance improvements in cycling and rising urea levels suggest that excessive physiological strain may be associated with constrained adaptation. Accordingly, tracking urea over time may provide useful information to prevent surpassing an athlete’s maximal tolerable load threshold. However, the substantial inter-individual variability observed confirms that isolated biomarker readings—urea or CK—are insufficient for diagnosing excessive fatigue. Their value lies in longitudinal monitoring, particularly when elevations persist across consecutive tests.

Further research is required to establish individualized interpretive models that align biomarker dynamics with training-load responses in a more precise and athlete-specific manner.

## Data Availability

The raw data supporting the conclusions of this article will be made available by the authors, without undue reservation.
